# CONGENITAL BATHING TRUNK NEVUS WITH AMBIGUOUS GENITALIA: A RARE COINCIDENCE

**DOI:** 10.4103/0019-5154.55649

**Published:** 2009

**Authors:** Devi Basanti, Mohanty Prasenjeet, Patro Nibedita, Panda Maitreyee, Swain Basanta

**Affiliations:** *From the Department of Skin & VD, SCB Medical College, Cuttack, India.*; 1 *From the Department of Radiology, SCB Medical College, Cuttack, India. E-mail: basanti1953@yahoo.co.in*

Sir,

A two-and-a-half-month-old infant born out of non-consanguinous marriage by normal vaginal delivery presented with a pigmented patch involving the trunk and whole right leg [Figure 1] since birth. The surface was rugose, leathery, and nodular with no hairs. Multiple satellite lesions of varying sizes were present all over the body. The right leg was thinner as compared to the left leg. External genitalia appeared to be enlarged labia majora with a deep nodular tissue of size approximately 1 × 1.5 cm on right side. On retracting the labia majora, it revealed labia minora, clitoris, and vaginal opening [Figure 2]. The examination of other systems were normal. There was no history of any seizures or focal neurological deficit. The infant was otherwise healthy with adequate feeding. The clinical diagnosis of congenital bathing trunk nevus was made and the biopsy findings were suggestive of congenital melanocytic nevus. The computerized tomographic scan of head and neck showed no abnormalities. The sonography of abdomen and pelvis revealed absent uterus and ovary. Before some higher investigations to rule out associated abnormalities and to confirm the genotypic sex of the infant could have been done, we lost the patient to follow up.

**Figure 1:**
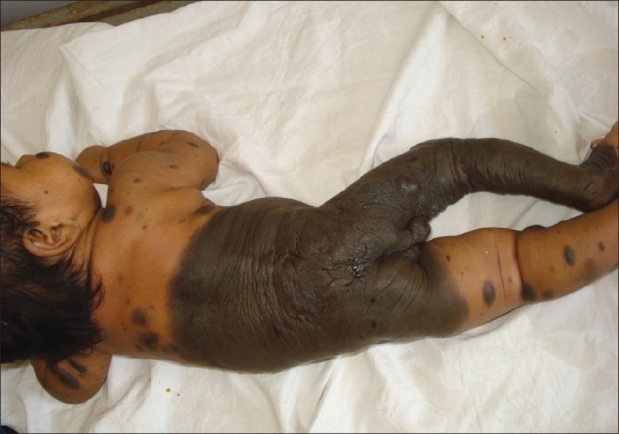
Deeply pigmented patch over lower trunk and right leg with multiple small satellite lesions

**Figure 2:**
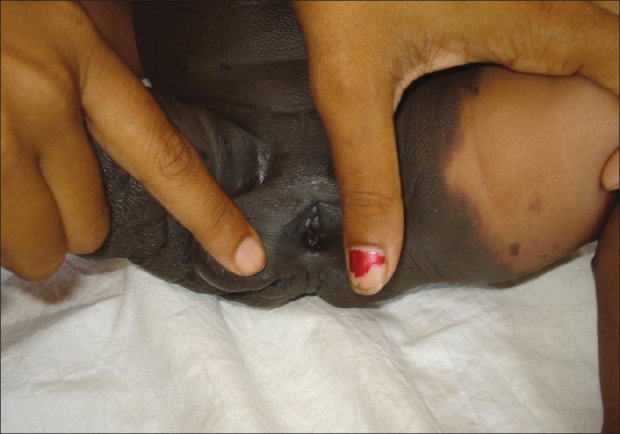
Labia minora, clitoris, and vaginal opening with enlarged labia majora

Congenital bathing trunk nevus is a congenital melanocytic nevus, also known as garment nevus or giant hairy nevus. The incidence is rare around 1 in 500,000 newborns.[1] It is obvious at birth and these lesions have diameters more than 20 cm. It is commonly present in the lower back and thigh areas. Multiple smaller congenital nevi called *satellite nevi* are commonly associated. The surface of the nevus is usually rugose or warty and nodular with large terminal hairs, which become more prominent as the infant grows toward puberty. These have an increased malignant potential. Various associated abnormalities are reported with it, the most common being leptomeningeal melanocytosis. Other common associated abnormalities are spina bifida, meningocoele and club foot and hypertrophy or atrophy of a limb involved by the nevus.[2] Hamartomas such as vascular nevi, lipomas, or von Recklinghausen's disease may be found.[2] Other associated tumors are schwannoma, rhabdomyosarcoma, neuroid tumor, nevus sebaceous, mastocytoma, etc.[1] Retrolumbar subcutaneous ependymoma has also been reported with giant bathing trunk nevus.[3]

Complete androgen insensitivity syndrome has been described as a X-linked recessive disorder.[4] The child is genotypically male but phenotypically female with a blind vaginal pouch, absence of uterus and ovary, and presence of testis. The child develops a female habitus at puberty with breast development but scanty pubic and axillary hair and primary amenorrhoea. There is increased levels of luteinizing hormone, testosterone, and estradiol. Follicle stimulating hormone is normal or increased and there is resistance to androgenic and metabolic effects of testosterone. In our patient, the clinical features can be correlated with this syndrome, but due to lack of investigations the diagnosis could not be established.

We report this case of congenital bathing trunk nevus associated with multiple smaller satellite nevi, atrophy of the right leg involved by the nevus, and a rare coincidental finding of ambiguous genitalia, which has never been reported earlier.

## References

[1] Grichnik JM, Rhodes AR, Sober AJ, Wolff K, Goldsmith LA, Katz SI, Gilchrest BA, Paller AS, Lefell DJ (2008). Benign Neoplasias and Hyperplasias of Melanocytes. Fitzpatrick's Dermatology in General Medicine.

[2] Mackie RM, Burns T, Breathnach S, Cox N, Griffiths C (2004). Disorders of the Cutaneous Melanocyte. Rook's Textbook of Dermatology.

[3] Bourlond J, Bourlond A, Rousseau C (1994). Retrolumbar subcutaneous ependymoma and giant bathing-trunk nevocellular nevus. Int J Dermatol.

[4] Achermann JC, Hughes LA, Kronenberg HM, Melmed S, Polonsky KS, Larsen PR (2008). Disorders of sex development. Williams Textbook of Endocrinology.

